# Influence of a Varying State of the Price of Provisions on General Disease and Mortality

**Published:** 1844-11

**Authors:** 


*Influence of a Varying State of the Price of Provisions on General Disease and Mortality.*—M. Mélier investigates with philosophic spirit this important medico-political question. The statistical documents he has collected, and the tables into which his figures are thrown, bear out the following inferences:

1. The mortality of a country is influenced by the price of corn and bread.
2. This influence was extremely marked formerly, and is less so at present.
3. The diminution of this influence has been gradual, and various causes have contributed to this result.
4. The cultivation of the potatoe is one of the chief of these.
5. The question is one of morality as well as of hygiene, for it is demonstrated that crime increases with the dearness of provisions.

It is inferable too, the author considers, from his inquiries, that, in a well-organized state of society, provisions tend constantly to increase in abundance; this tendency is more marked than that of the population to increase—'a powerful argument against the theory of Malthus.'

*British and Foreign Med. Review.*

